# Why Healthcare Providers Should Focus on the Fertility of AYA Cancer Survivors: It’s Not Too Late!

**DOI:** 10.3389/fonc.2013.00248

**Published:** 2013-10-07

**Authors:** Devin Murphy, Etan Orgel, Amanda Termuhlen, Susan Shannon, Krista Warren, Gwendolyn P. Quinn

**Affiliations:** ^1^Jonathan Jaques Children’s Cancer Center, Miller Children’s Hospital, Long Beach, CA, USA; ^2^Department of Pediatrics, Keck School of Medicine, University of Southern California, Los Angeles, CA, USA; ^3^Health Outcomes and Behavior Program, Moffitt Cancer Center, Tampa, FL, USA; ^4^College of Medicine, University of South Florida, Tampa, FL, USA

**Keywords:** fertility, survivorship, adolescent and young adult, oncology, discussion

## Abstract

Reproductive health among cancer survivors is an important quality of life issue. Certain cancer therapies have known fertility risks. There is an existing cohort of adolescents and young adults (AYA) cancer survivors that, seen less frequently in clinical care settings than active patients, are likely not having discussions of fertility and other reproductive health issues. A survivor or healthcare provider can easily assume that the window of opportunity for fertility preservation has passed, however emerging research has shown this may not be the case. Recent data demonstrates a close relationship between fertility and other late effects to conclude that ongoing assessment during survivorship is warranted. Some fertility preservation procedures have also been shown to mitigate common late effects. This review explores the link between late effects from treatment and common comorbidities from infertility, which may exacerbate these late effects. This review also highlights the relevance of fertility discussions in the AYA survivorship population.

## Introduction

Reproductive potential and other reproductive health concerns among cancer survivors is an important quality of life issue. Certain cancer therapies, particularly alkylating chemotherapy agents and radiation, have known fertility risks. Fertility issues within oncology have ignited a robust research focus that explores physiologic and psychological late effects, advancing technologies that reduce the risk of becoming infertile in the future, and broader reproductive health concerns such as appropriate contraception and the HPV vaccine (1–4). Sustained infertility may develop in 50–95% of cancer survivors, largely in patients who have undergone high-dose chemotherapeutic conditioning regimens for bone marrow transplants (5–7). However, there are fertility preservation options available to adolescents and young adults (AYA) at multiple time points during the course of cancer care. The most efficacious time to pursue such options is before starting treatment, though the perceived need to start treatment immediately often hinders these options.

There are heterogeneous reports exploring the rates of discussion of fertility. Ranging from 34 to 70% (8–11), whether or not a patient receives information is based on a variety of factors, many of which are outside the individual’s. It is clear however, that the majority of cancer survivors did not receive information on fertility prior to treatment.

Adolescents and young adults cancer survivors have distinct developmental needs. While many adolescents without a history of cancer spend these formative years affirming their unique identity, survivors are on a journey toward “normal,” as they have experienced significant disruption in identity formation (12). Engaging in social and romantic relationships is a healthy developmental milestone of any AYA, wherein sexuality is explored and future plans to marry and have children are considered (13).

With attention to fertility gaining more attention in pediatric oncology settings, discussions are improving (11). That is significant progress for newly diagnosed patients, and we anticipate this trend will continue. However, there is an existing cohort of AYA cancer survivors that, seen less frequently in clinical care settings than active patients, are likely not having discussions of fertility and other reproductive health issues. The Children’s Oncology Group (COG) has issued long-term follow-up guidelines that detail the management of pertinent late effects which include referrals to reproductive specialists (14), but lack specifying what fertility options may be valuable during survivorship. COG also recently assembled task forces to develop guidelines for male and female survivors and reproductive health but do not address how the effects of infertility may compound existing late effects (15–17).

This AYA survivorship cohort is growing (16) but survival does not come without a cost. AYA cancer survivors have been found to have poorer physical and psychosocial outcomes than their healthy peers, thus warranting close monitoring. AYA cancer patients diagnosed before the American Academy of Pediatrics (18) and the American Society of Clinical Oncology (19) published guidelines on fertility preservation in pediatric cancer patients in 2008 and 2006, respectively, were not likely to receive reproductive health information prior to treatment. Does that mean the window of opportunity to make referrals to reproductive specialists has passed? This review explores the relevance of ongoing fertility discussions in the AYA survivorship population due to the interrelatedness of common late effects from treatment, and common comorbidities from infertility, which may exacerbate or be concealed within these late effects. We also discuss fertility preservation options that are available to AYAs post-treatment that may also treat some specific late effects.

## The Importance of Reproductive Health Monitoring

**Figure 1:**
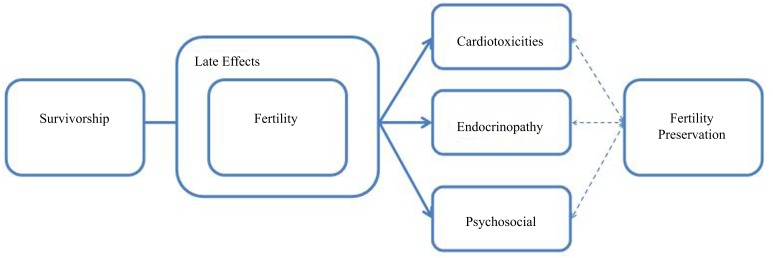
**Relationship of fertility and late effects in survivorship**.

There are likely misconceptions regarding AYA cancer survivors’ quality of life concerns. There is an overshadowing focus on survivors’ disease status and the assumption that cancer survivors are less interested in sex and reproduction than their healthy peers. In reality, research indicates adolescents with a chronic illness are at least as sexually active as their healthy counterparts (20, 21). The sentiments of the medical community, combined with cultural taboos of AYA sexuality present barriers to conveying medically imperative information to survivors regarding fertility, sex education, contraception, and risks of sexually transmitted infections (STIs). There is a distinct interrelatedness among existing late effects experienced during survivorship and fertility preservation outcomes after treatment (Figure 1). Fertility preservation options that may mitigate treatment-induced late effects are highlighted later in this review.

## Physiological Effects of Infertility

There are unique relationships among infertility and the late effects of cardiotoxicity, bone health, and other endocrine disorders. It is important for healthcare providers (HCP) to be aware of the potential for infertility to exacerbate these late effects. At the same time, HCP must also be aware of the risk for infertility symptoms to go untreated due to a focus on other common physiological late effects. We discuss late effects that HCP routinely assess, and the similarity of these symptoms within infertile persons that may go overlooked.

### Cardiotoxicity

It is well documented that survivors are at risk for cardiovascular disease and mortality due to treatment alone (22–24). Females have an increased susceptibility to cardiotoxicities during survivorship (25). Radiation therapy and certain chemotherapeutic agents utilized in the treatment of pediatric cancer are frequently linked to cardiac dysfunction in survivors (26). Long-term effects of radiation on the cardiovascular system can include premature coronary artery disease, constrictive pericarditis, and pericardial effusions (27). Further, the chemotherapeutic classes of Anthracyclines are agents generally associated with cardiac sequelae such as acute-arrhythmias, hypotension, and a decrease in cardiac function, which can lead to congestive heart failure (28).

Infertility may aggravate these existing cardiovascular impairments. Females who experience premature ovarian failure (POF) as a result of treatment are at risk of cardiovascular disease due to the early loss of estrogen (29, 30). Estrogen has been shown to maintain healthy blood pressure and plasma fibrinogen levels, a risk factor of cardiovascular disease. Estrogen prevents white blood cells from adhering to the lining of blood vessels, therefore patients with normal estrogen levels have a reduced risk of developing atherosclerotic plaque (31, 32). Among men, testosterone serves as a cardioprotective hormone as well, and a significant decrease in testosterone, as is often experienced following chemotherapy, places patients at risk for cardiovascular disease (33). Low testosterone may also contribute to coronary artery constriction (34), which may increase survivors’ risk of atherosclerotic disease and stroke (35). Infertility can compound the cardiovascular risk existing from treatment, therefore there is an increased need for specialized attention to manage survivors and could necessitate changes in the interventions prescribed.

### Bone health

Bone health may also be affected by both infertility and cancer treatment, and should be regularly assessed at follow-up visits. Reduction in bone health quality from treatment can occur in a variety of ways. Many commonly utilized chemotherapies, such as corticosteroids, antimetabolites, anthracyclines, and alkylating agents have been shown to adversely impact bone mineral density (BMD) through creating an imbalance in bone metabolism with increased bone resorption and decreased bone formation (36–38). These agents are directly toxic to osteoblast maturation, inhibit bone promoting growth factors, and promote osteoclast activity. The question of bone health is particularly problematic in a pediatric patient population as an individual’s peak bone mass for life is attained in late adolescence (39). The chemotherapy necessary for cure therefore coincides and interferes with this vital time period for bone formation with lifelong implications.

Gonads in both males and females secrete hormones that facilitate bone metabolism (40). There is a clear association between skeletal health and gonadal function in survivors (41) that highlights the need to address reproductive health in this population to maximize potential bone density even long after therapy is completed. Recent research has examined the relationship between skeletal health and ovarian function, which exhibits a cyclical association. Females with POF often have low BMD increasing the risk of fracture and delayed healing (42, 43). Specifically, patients with POF caused by chemotherapy present with rapid bone mineral loss (44). The cyclical relationship is exhibited in the exacerbation of vasomotor symptoms such as hot flashes and night sweats related to infertility by low BMD (45). Because estrogen helps protect against the destruction of osteoblasts, the decrease of estrogen due to infertility has potential to cause significant bone health issues for survivors. The rapid loss of estrogen in women experiencing POF, which acts as a transition period to menopause, accelerates the rate of BMD loss, and patients who have poor bone health due to treatment-induced infertility may experience further deterioration. This places even young survivors at an increased risk for osteopenia and osteoporosis.

In males, there is a unique, reciprocal relationship between bone health and fertility. Evidence for this can be found in adults with liver cancer; in those with impaired gonadal function and osteoporosis, liver transplantation was found to have restorative action on both gonad function and bone health (46). Conversely, low BMD can induce infertility in males. Osteocalcin promotes production of testosterone in the testes, and low osteocalcin therefore impacts testosterone level as well (47). Assessment of fertility function by providers during visits is therefore one key determinant of poor bone health. Similarly, as BMD is often routinely monitored in AYA survivors, those with poor bone health, providers should also routinely assess fertility and testosterone levels as risk factors for infertility. The strong association between fertility and bone health in males and females supports the current recommendations for, providers to assess gonadal function during follow-up visits.

### Endocrine late effects

Perhaps the strongest relationship warranting close assessment is that of infertility and endocrine late effects. Up to 50% of survivors have reported effects such as hypopituitarism, precocious puberty, and adrenal insufficiency (48). Endocrine disturbances are largely attributed to the location of the cancer, the class and dose of chemotherapy, the amount, location and type of radiation, and the length and time from treatment, similarly to the risk of infertility. Survivors treated at a young age or with radiation to the head, neck, or spine are at the greatest risk for developing endocrinopathies (49). In fact, up to 90% of childhood brain tumor survivors have been reported to have some evidence of growth hormone deficiency at a median of 4 years post-treatment (50). Thyroid deficiency may cause fatigue, weight gain, and depressed mood which mimic frequent symptoms of infertility. Early recognition and treatment of these conditions is extremely important since they can have a significant influence on optimal growth and development, cognition and progression of pubertal maturity, but should not be assessed in isolation from fertility status. Endocrine late effects include those of reproductive health. Cancer treatment such as radiation and alkylating agents like cyclophosphamide and procarbazine used for solid tumors may damage endocrine organs that have a high cell division rated, preventing the function of gonadotropin-releasing hormones (51). The affected pituitary can fail to produce hormones that stimulate testosterone and estrogen production (52). Impaired hormonal metabolism affects gonadal function and the ability to produce sperm and release eggs. Further, treatment-induced hyperprolactinemia can affect the reproductive system in males and females. In females, high prolactin also causes galactorrhea (breast milk production in a non-breast feeding person) and absent or irregular menses (53). In males, hyperprolactinemia can trigger galactorrhea and low testosterone resulting in decreased libido (54). Hyperprolactinemia can be caused by radiation to the hypothalamus gland (55).

In females, chemotherapy and radiation have varying risks on the reproductive organs as well as the endocrine system that regulates hormones within these organs. Females are particularly at risk of infertility due to cancer treatment as females are born with a finite number of non-replicating oocytes. Furthermore, luteinizing hormones stimulate theca cells to produce a steroid hormone that is converted to sex hormones in the granulosa cell which is stimulated by follicular stimulating hormone (FSH) (56). This process mature oocytes. Damage to theca and granulose cells from alkylating chemotherapeutic agents can prevent this maturation process and cause premature menopause (57). It is difficult to predict when POF will occur, as some reports have shown this immediately after administration of treatment, while others have reported a significant delay (15, 58). Endocrine function not only plays a role in maintaining ovarian function, but also in predicting it. Assessing direct hormone products of the ovary such as FSH, Inhibin B, and anti-Mullerian hormone (AMH), can show how the ovarian is functioning. Measuring AMH is useful in the detection of early growing follicles, which may be a better predictor of ovarian reserve (59).

Men may experience comparable menopausal symptoms during andropause such as osteoporosis and sexual dysfunction as a result of a dramatic decrease in testosterone (60). Andropause occurs naturally among aging males, and technology can help predict if a male is at risk for premature andropause. However, it is difficult to predict when an AYA male will experience these symptoms after cancer therapy. Screening tests such as the Androgen Deficiency in Aging Males (ADAM) self-report questionnaire, as well as evaluating testosterone serum levels are valuable in monitoring the likelihood of andropause (61). It is difficult to assess how long after the completion of treatment an AYA will enter permanent menopause/andropause, which may still occur naturally at the anticipated time point later in life, necessitating an ongoing assessment plan. Hormone therapy is the treatment for many endocrine disorders, which may also alleviate negative symptoms of infertility such as abnormal or painful periods in females, skin changes, and hair growth. Conversely, treatment for infertility may reduce the impact of endocrine disorder symptoms. The interrelationship among endocrine dysfunction from cancer treatment and reproductive health issues from cancer treatment provides evidence for examination of their combined potential to impact AYA survivors. Knowledge and awareness of late effects along with clinical vigilance and early intervention is crucial for this unique population in order to reduce the impact and morbidity of these late effects.

## Psychological Effects of Infertility

Male and female cancer survivors can be burdened with emotional, social, and psychological consequences as a result of infertility. AYA survivors have experienced a dramatic acceleration of life’s challenges, beginning with confronting their own mortality. Survivorship comes with a new set of complications, negotiations, and coping skills. Adolescent cancer patients have been shown to have long-term goals that include childbearing and parenthood, as well as informational needs for fertility preservation (62). A diagnosis of cancer does not derail these goals. As a result of infertility, some survivors have reported low self-esteem, regret for not questioning infertility risks, guilt for current partner, or fear of never finding a partner (63).

Other barriers may prevent the assessment of reproductive potential aside from solely physician awareness. Males may be uniquely overlooked regarding the psychosocial effects of survivorship. Male AYA survivors have been noted to have issues with body image and appearance, masculinity and sexuality, cynical about planning for future parenthood, and afraid of transmitting cancer to a partner (64). Perceptions of male stoicism and lack of concern about appearances may prevent these issues from being assessed due to a fear of embarrassment for both the patient and provider. Additionally, there are financial costs associated with assessment of and treatment for fertility preservation that are currently not always covered by health insurance. To this end, the potential financial burden to parents and patients may also inhibit providers from discussions.

The existing AYA survivorship cohort seen for follow-up care is in a unique transitional period, not only the transition of patient to survivor, but also the normative developmental transition of adolescent to adult. Past goals are now seen as capable of becoming actualized in adulthood, including goals for parenthood; an unknown fertility status may heighten distress and anxiety (1). Zebrack et al. found that of 32 childhood cancer survivors, the majority placed a high value on future parenthood, however 60% had an unknown fertility status (1). An unknown fertility status has potential to magnify psychosocial issues arising from the cancer experience as well as create unique stressors as a result of the cancer experience. For example, Halliday et al. reports that young female survivors felt “rushed” to enter parenthood because time spent on treatment had disrupted formative reproductive years (65). Halliday goes on to explain feelings of “otherness” felt by female survivors with an unknown fertility status, in that rejoining the young adult social cohort creates a sense of devalue and divergence from the norm from those who did not experience cancer and assume a fertile capacity (65, 66). Infertility, whether confirmed, perceived, or questioned, invokes a variety of coping mechanisms such as information-seeking and anticipatory grief that can be supported and directed with appropriate psychosocial care.

## Fertility Options for Survivors

Fertility can be compromised in different ways for males and females depending on the initial disease site, stage, age of patient, therapeutic treatment regimen, and condition of the patient. Alkylating agents within some chemotherapies do not target specific cells, and therefore affect all cells, especially those that divide rapidly such as cancer cells in the bone marrow. Other cellular activity can be impacted such as also oogoniums in pre-pubertal females, primary oocytes, and sperm cells, as well as theca and granulose cells in ovarian follicles that mature oocytes (67–69). Because alkylating agents have high potential to destroy these important reproductive cells, pursuing fertility preservation prior to treatment is most advantageous and carries the greatest likelihood to restore reproductive potential. HCP may believe this damage is irreparable and unavoidable, and elect to avoid reproductive health discussions during survivorship care. However, there is evidence that pursuing fertility preservation options even after treatment, and even after confirmed infertility, shows potential to regain reproductive function. Successful pregnancies after ovarian tissue cryopreservation followed by transplantation have been reported in some human studies (70–74). Gosiengfiao et al performed a unilateral oophorectomy on a 9-year-old female who had finished treatment for rhabdomyosarcoma, which included alkylating agents such as cyclophosphamide. Examination of the re-implanted tissue found viable primordial follicles despite aggressive treatment (75). Additionally, Meirow et al reported a live birth resulting from ovarian tissue cryopreserved after treatment for Non-Hodgkin Lymphoma and confirmed ovarian failure for 2 years in a 28-year-old survivor (72).

Male survivors who did not undergo a fertility-sparing procedure before treatment also have options post-treatment if fertility is impaired. Due to the rapid regeneration of sperm, recovery of reproductive potential in males after treatment with non-akylating chemotherapeutic agents is higher than with alkylating agents (76). Naysmith et al. confirmed that fertilization could be achieved via testicular biopsy of a male testicular cancer survivor 8 years post-treatment, who was confirmed to be azoospermic (77). The options for survivors who are interested in a future pregnancy are expanding as rapidly as the science behind it. Men with confirmed infertility years off treatment can still take advantage of testicular sperm extraction (TESE) and Intracytoplasmic sperm injection (ICSI) methods where minimal sperm can be used to achieve a pregnancy (70). Physicians should take advantage of the availability and expertise of reproductive specialists who can consult with the patient and determine the eligibility for procedures.

## Other Benefits of Fertility Preservation

In addition to achieving pregnancy, some fertility preservation options may offset other late effects experienced as a result of treatment. Recent experimental trials have examined the benefits of re-implantation of ovarian tissue in females for purposes other than achieving a pregnancy. Transplantation of cryopreserved ovarian tissue has shown to be a potential method for recovery of ovarian function (78, 79), which carries a variety of benefits by staving off symptoms of menopause. Ovarian tissue transplantation has recently been shown to restore endocrine function in young women after cancer treatment (80, 81). Oktay et al. transplanted cryopreserved ovarian tissue that resulted in decreased FSH and LH levels as well as stabilizing estradiol levels (81). This took approximately 10 weeks after transplantation. Other patients resumed normal menstrual cycles and hormone levels about 5 months after transplantation (82, 83). Males have been shown to resume testosterone production after re-implantation of cryopreserved testicular tissue (84). Though no study has examined testicular tissue taken after cancer treatment, some infertility treatments such as TESE/ICSI show promise that even after treatment testicular tissue may be viable and could offer endocrine and cardiac benefits similarly to ovarian tissue transplantation.

## Conclusion

It is important to keep up with the fast-paced technologies of fertility treatments, as it is to stay abreast of the latest treatment regimens. Active treatment is being re-shaped by the push to discuss fertility preservation options prior to treatment, which is a result of the needs expressed by the ever-expanding, vocal survivorship cohort that exists today. What’s now needed is a way to bridge these two worlds and inform survivors that the window of opportunity for future parenthood, reversing POF and other late affects may not be gone. Long-term follow-up guidelines should incorporate the benefits of discussing fertility and fertility preservation options so that HCP can ensure comprehensive care and long-term quality of life.

## Conflict of Interest Statement

The authors declare that the research was conducted in the absence of any commercial or financial relationships that could be construed as a potential conflict of interest.
